# Role of supersaturated Al-C phases in mechanical properties of Al/fullerene composites

**DOI:** 10.1038/s41598-021-92551-y

**Published:** 2021-06-23

**Authors:** Seungjin Nam, Sooun Lee, Aeran Roh, Hansol Son, Miso Kim, Hyunjoo Choi

**Affiliations:** 1 School of Materials Science and Engineering, Kookmin University, 77, Jeongneung-ro, Seongbuk-gu, Seoul, 02707 Republic of Korea; 2 AI Metamaterial Research Team, Korea Research Institute of Standards and Science (KRISS), 267 Gajeong-ro, Yuseong-gu, Daejeon, 34113 Republic of Korea; 3 School of Advanced Materials Science and Engineering, Sungkyunkwan University, Suwon, 16419 Republic of Korea

**Keywords:** Materials science, Nanoscience and technology

## Abstract

We investigated the reinforcing effect of supersaturated Al-C phases on the mechanical properties of Al/C_60_ composites produced via powder metallurgy followed by thermal treatment. We controlled the fractions of C_60_-fullerenes, nano-scale carbides, and Al-C supersaturated phases in the Al/C_60_ composites by adjusting the heat-treatment temperature and duration. Furthermore, we examined the contribution of each phase on the elastic and plastic behavior of the composites using scanning acoustic microscopy (SAM) and hardness measurements. After heat treatment, a supersaturated Al-C phase and an Al carbide were formed in the Al/C composites by decomposition of individually dispersed C_60_. This led to enhancement of the hardness and elastic modulus of the Al/C composites heat-treated at 450 and 500 °C, while these properties were reduced in the 650 °C heat-treated composite. Notably, the 500 °C heat-treated composites showed significantly high hardness and elastic modulus (approximately 250 Hv and 77.8 GPa, respectively) owing to the substantially large contribution of the supersaturated Al-C phases, which was theoretically calculated to be 851 GPa/vol% and 227 GPa/vol%, respectively. This is possibly because the well-dispersed C in the atomic scale changed the elastic bonding characteristics of the metallic bonds between the Al atoms.

## Introduction

The development of lightweight energy-efficient materials with high-level performance is a major concern in several industrial fields for compliance with the increasingly demanding global environment regulation trends while maintaining production. Aluminum is the most abundant metal on earth, and it is also the fourth most electrically/thermally conductive and the second lightest metal (2.7 g/cm^3^ in density) among metallic structure materials^1–3^. It has significant uses in structural and functional components in the automotive, biomedical, and electronic industries. Demand for aluminum is moreover expected to increase 1.5-fold within the next 4–5 years^4^.

Carbon has been one of the key components to greatly alter properties of metals from the beginning of human history. For example, when carbon is dissolved in liquid iron, it produces Fe–C alloys after solidification with various strength ranges from low–yield (with ferrite) to high–yield stress (with martensite)^5^. When carbon atoms do not have enough time to diffuse in sufficient quantities to form compounds (Fe_3_C in the Fe–C alloy system), α-Fe (i.e., ferrite) is supersaturated with carbon atoms, forming a highly strained body-centered tetragonal phase (i.e., martensite), which is the primary strengthening mechanism of dual-phases and martensitic steels^6^. However, metal-C alloy systems other than Fe–C alloys have not been widely reported, because, unlike iron, other anionic elements, such as carbon (C), nitrogen (N), or oxygen (O), are not considered to be alloyed in most metal systems since their solubilities in the solid state are negligible.

Carbon is rather used as a reinforcement of other metallic materials including aluminum^7,8^. In particular, nanocarbon materials, such as fullerene, multi-walled carbon nanotubes (MWCNTs), and graphene, have been introduced to metallic materials, providing them with excellent mechanical and functional properties, as well as low density; the carbon nano-reinforcements dramatically improve the properties of Al-matrix nanocomposites by these reinforcing effects^9–14^. The true tensile strength of Al/CNT (5 vol%) composites and pure Al has been measured at 194 MPa and 85 MPa, respectively^11^. Furthermore, Shin et al.^12^ reported an outstanding reinforcing effect of 0.7 vol% few-layered graphene (FLG) on Al-matrix nanocomposites, produced via powder metallurgy; the evaluated yield strength of monolithic Al and Al/FLG nanocomposites was 262 and 440 MPa, respectively. Even though nano-reinforcements significantly strengthen the matrix even at low contents, the reinforcing effect of the nanocarbon materials on Al-matrix nanocomposites could not reach the expected level because of the poor wettability between the matrix and nanocarbon reinforcement^15–17^. Moreover, nano-scale carbides may be formed due to the reaction between Al and C decomposed from the reinforcement, when the fabrication process is performed at high temperatures^18,19^.

In our previous study, carbon that was thermally decomposed from individually dispersed fullerenes was intercalated into the Al interstitial sties to form Al-C phases after annealing, rather than aluminum carbides^20^. This may happen under the special circumstance where the thermal energy for chemical decomposition of fullerenes is sufficiently lower than that for carbide formation because of the small radius of fullerenes. Interestingly, the formation of Al-C phases results in significant enhancement of the yield strength and damping capacity^21,22^; the annealed Al/fullerene composite exhibits a yield strength of approximately 1 GPa and greatly enhanced damping ability compared to its non-treated counterpart. The Al-C phases may act as nano-scale domains with a distorted lattice structure in the Al matrix^20^, greatly hindering dislocation movement and altering bonding characteristics. However, a systematic study has not been conducted on the role of fullerene, aluminum carbides, or these unknown Al-C phases on the elastic and plastic behaviors of Al-based materials.

**Figure 1:**
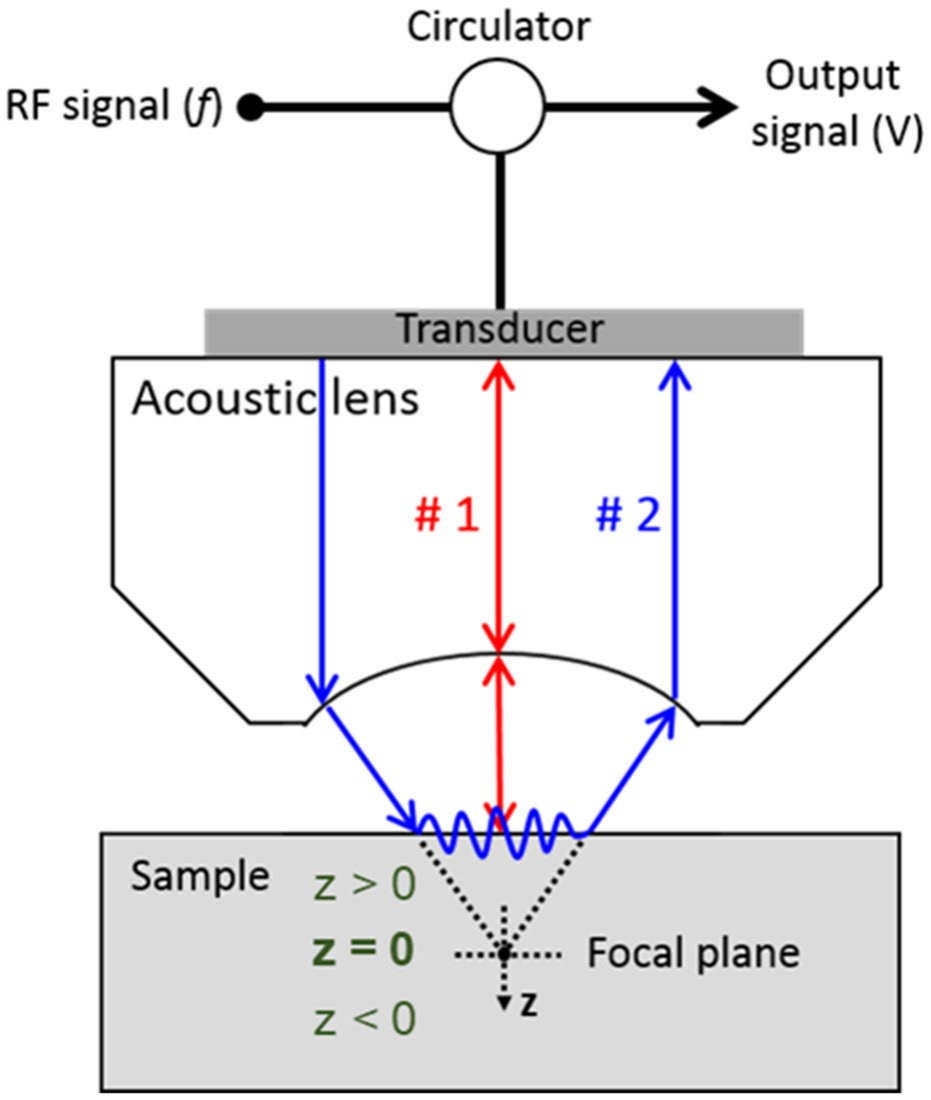
Schematic illustration of working principle of scanning acoustic microscopy.

Scanning acoustic microscopy (SAM) is a powerful nondestructive technique for the study of the physical properties of materials, where ultrasonic waves are utilized as probes equivalent to light waves in optical microscopy^23^. SAM can provide a wealth of information that is not available from optical microscopy as acoustic images are based on physical characteristics other than the optical refractive index. In an acoustic microscope, a piezoelectric transducer transmits acoustic waves through an acoustic lens, and these waves converge at the focal plane of the lens and follow one of two wave paths. Following the first path, the waves propagate on the interface between the specimen and the coupling medium (e.g., water), and are reflected back to the lens (line #1 in Fig. 1). In the other path, the waves are refracted at certain angles, exciting leaky surface acoustic (i.e., Rayleigh) waves along the specimen surface (line #2 in Fig. 1). The interference between the reflected and radiated waves results in periodic oscillations of the transducer voltage *V* as a function of the vertical direction *z*, which is known as the *V*(*z*) curve. Since sound waves interact with the elastic properties of the materials, the *V*(*z*) curve characteristics (e.g., periodicity) are related to the unique elastic properties of the specimen materials, such as elasticity and mass density. In this respect, SAM has actively been employed to determine the elastic constants of both bulk and thin-film materials^24–27^, as well as to evaluate adhesion between the substrates and the thin films^28,29^.

In this work, we applied the SAM technique to measure the phase velocity of the surface acoustic waves nondestructively, which, in turn, enabled determination of the elastic modulus of the Al/C composites containing variable contents of fullerenes, aluminum carbides, and Al-C phases. Furthermore, the study aims to systematically investigate the effect of newly explored phases, namely supersaturated Al-C phases, on the elastic modulus and hardness of Al/nano-C composites.

## Results

### Microstructural observation in Al/C composites from starting materials

**Figure 2:**
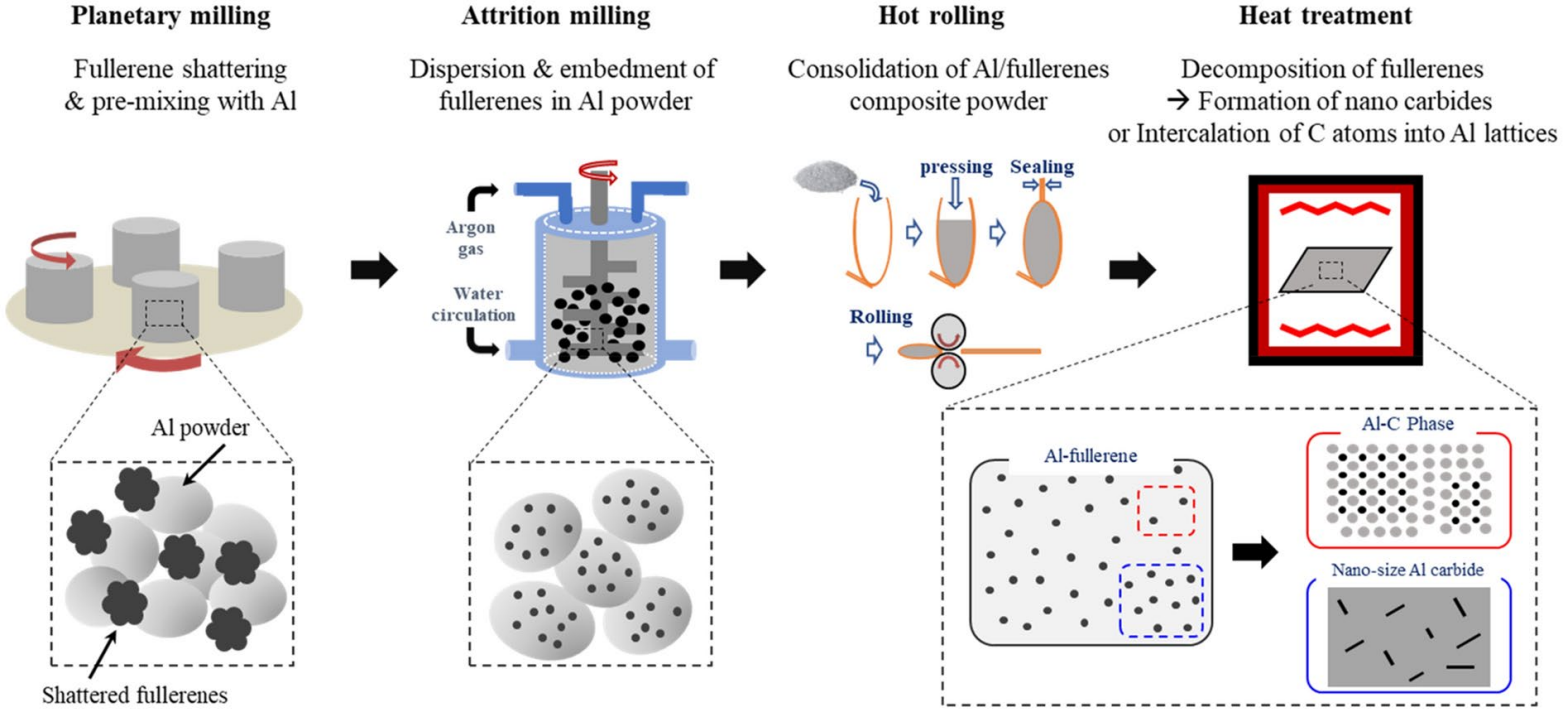
Schematic showing experimental procedure of Al-C nanocomposites.

**Figure 3:**
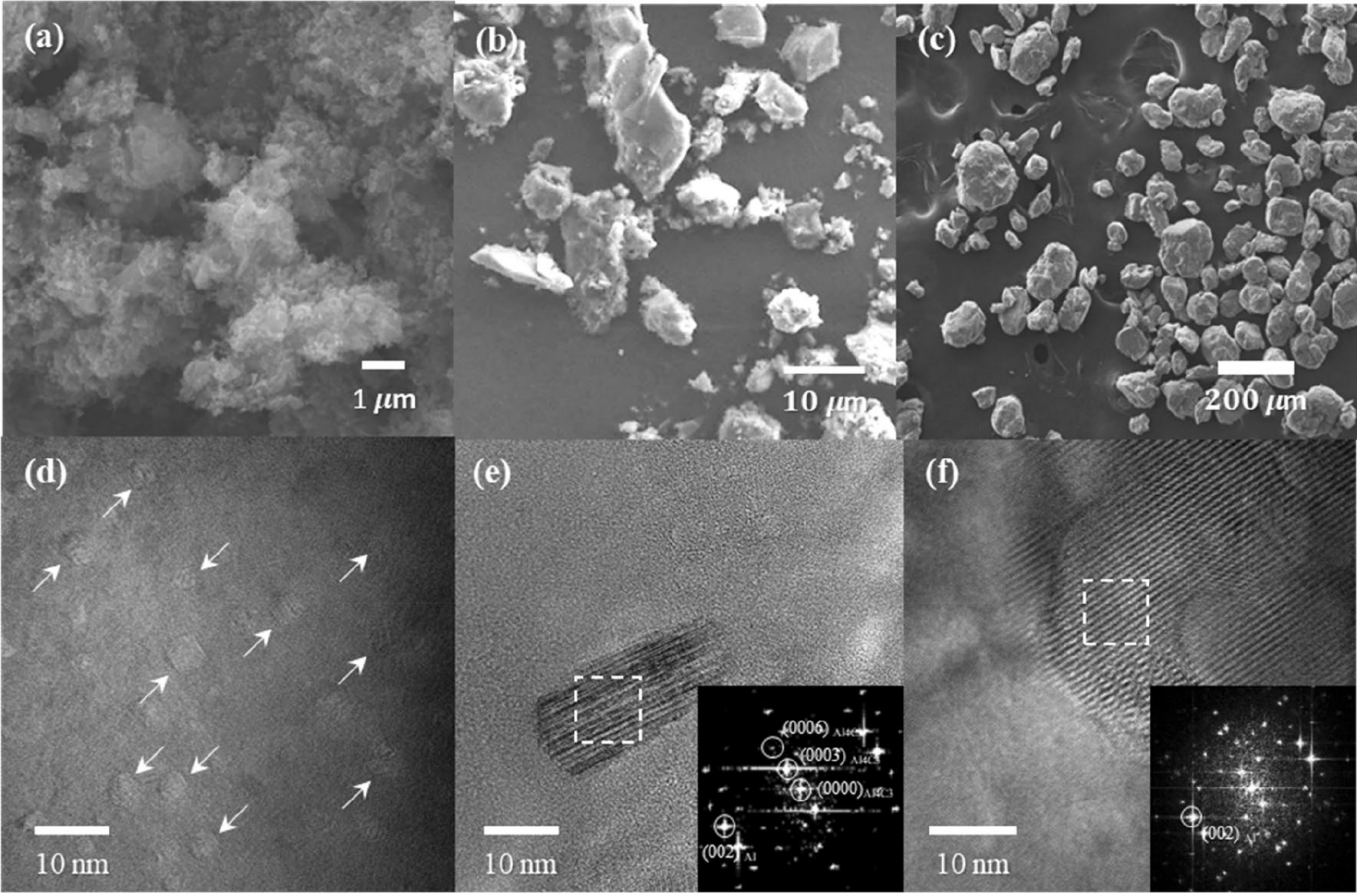
SEM images of (**a**) shattered fullerenes, (**b**) mixture of Al/fullerene powder, and (**c**) Al/fullerene composite powder, and TEM images of (**d**) fullerene dispersed in Al, (**e**) nano-sized Al carbide, and (**f**) Al-C phase. The inserted FFT patterns were obtained from the area marked by white squares.

In this study, Al/C composites were prepared via hot rolling from three-step ball-milled powders, followed by heat treatment, as described in Fig. 2. The microstructural evolution in the composites was observed during the procedure. Figure 3a–c shows scanning electron microscope (SEM) images of the ball-milled powder at each processing step; Fig. 3a reveals the shattered fullerenes, and Fig. 3b,c presents the pre-mixture of Al powder and fullerene and the ball-milled composite powder, respectively. During planetary milling of pristine fullerenes, the van der Waals bonds among fullerenes are weakened by ethyl alcohol, and the fullerene aggregates are disaggregated into tiny particles due to the impact energy of the balls. The shattered fullerenes are mixed with Al powder, as shown in Fig. 3b, and the hard fullerenes are embedded into soft Al powder during attrition milling (Fig. 3c). Figure 3d shows a transmission electron microscope (TEM) image of the as-rolled Al-fullerene composite, wherein the fullerenes are dispersed well in the Al matrix. Moreover, Fig. 3e,f presents TEM images of Al/C composites heat-treated at 500 °C for 12 h. After heat treatment, carbon atoms, decomposed from fullerenes, are formed from both, the nano-sized Al_4_C_3_ (Fig. 3e) and the supersaturated Al-C phases (Fig. 3f), as described in Fig. 2. The inserted Fast Fourier transform (FFT) patterns were obtained from the area marked by white squares. Generally, Al and C atoms tend to form compounds (i.e., Al carbides) instead of solid solutions at high temperatures because of the low formation free energy^17,18,30^. However, we recently reported the formation of meta-stable Al-C solid solution phases when carbon atoms are insufficient to form carbides^20^. The individually dispersed fullerenes can be easily decomposed at relatively low temperatures because of their small radius and high chemical potential, and the carbon atoms might fail to form carbides at these low temperatures and occupy interstices of the Al lattice^31^. Hence, Al-C phases could be formed by the intercalation of thermally decomposed C atoms into the interstitial sites of the Al matrix, resulting in an increase in the lattice parameters of Al^20^. This could be supported by the mixed moiré fringes produced by two lattices with a small misfit^21^, as shown in the inserted FFT pattern in Fig. 3g. In contrast, Al_4_C_3_ is a compound with a hexagonal structure (R-3 m space group) and has cell parameters of a = 0.3335 and c = 0.8542 nm. Due to their different structural characteristics, the Al-C phases can be distinguished from the Al_4_C_3_ phases by the inserted patterns in Fig. 3g,f.

### The evolution of phase and molecular structure after heat treatment

**Figure 4:**
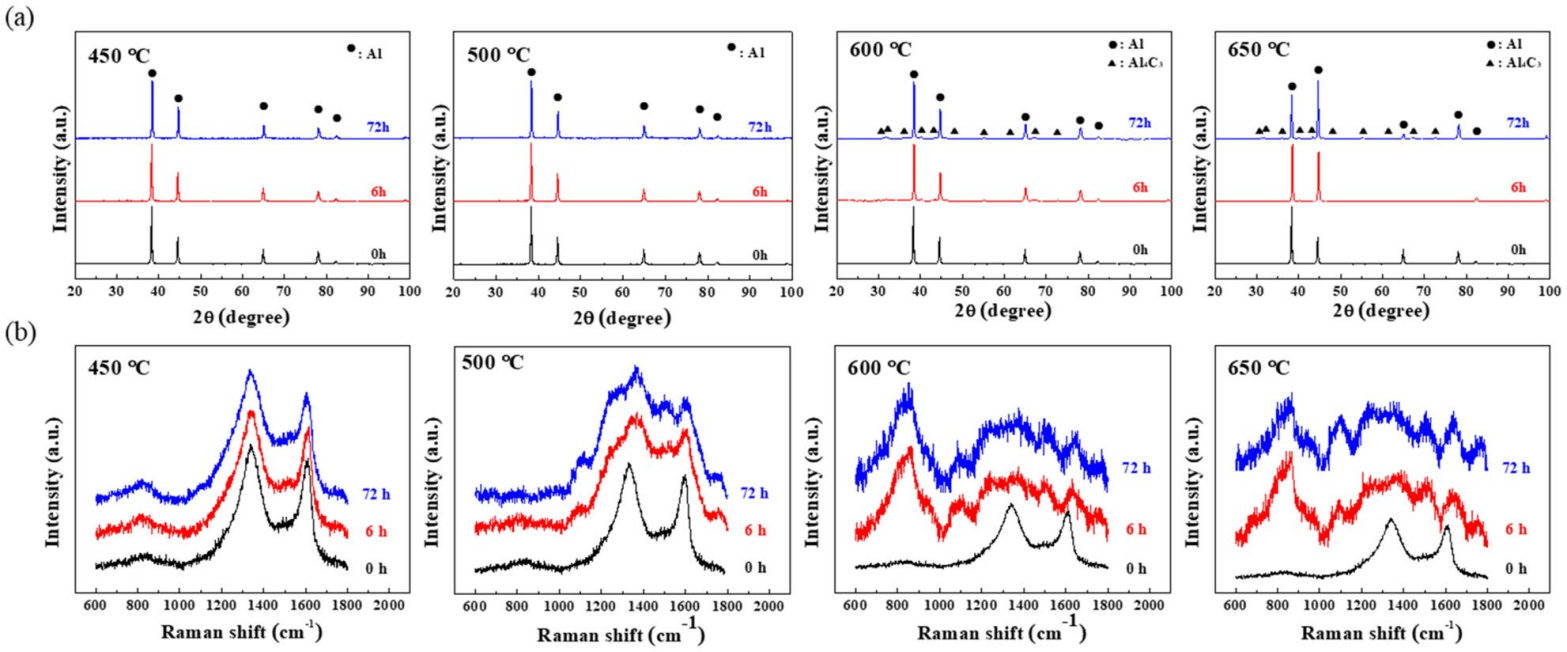
(**a**) XRD patterns and (**b**) Raman spectra of of Al/C60 nanocomposites after heat treatment at various temperatures for 6 and 72 h.

Figure 4a shows X-ray diffraction (XRD) patterns of the composites heat-treated at 450, 500, 600, and 650 °C for 0, 6, and 72 h. When the composites were heat-treated at 450 and 500 °C, the pattern reveals only peaks corresponding to the presence of Al without showing any peaks corresponding to Al_4_C_3_ regardless of the heat-treatment duration. However, the Al_4_C_3_ phase is detected in the Al/C composites after heat treatment at 600 and 650 °C, even for 6 h only. Under these conditions, carbon from the fullerene reacts with the matrix. Furthermore, lattice parameters of Al/C composites (4.08 Å) were calculated to have a slight difference as compared to those for monolithic Al (4.05 Å). On the other hand, the difference in lattice parameters among heat-treated composites was negligible (~ 0.01 Å). The relatively small volume fraction of Al-C phases in the composites may result in very weak intensity of the peaks originating from this phase in spite of the expanded lattice parameter of Al-C phase (4.25 Å)^20^.

The evolution of the molecular structure in fullerenes was investigated depending on the heat treatment conditions. Figure 4b exhibits Raman spectra of the as-rolled Al-fullerene composites and those heat-treated at 450, 500, 600, and 650 °C for 6 and 72 h. The as-rolled Al-fullerene composite reveals the D and G bands typically associated with defects and disorder of carbon (D band) and fullerenes (G band) at approximately 1340 and 1600 cm^−1^, respectively^32^. While the Al_4_C_3_ peak is present in the Al/C composite heat-treated at 450 °C, the spectra of composites heat-treated at 500 °C indicate partial destruction of the fullerenes’ molecular structure, but do not indicate the presence of Al_4_C_3_. Composites heat-treated at 600 and 650 °C exhibit a clear Al_4_C_3_ peak at 850 cm^−1^ as well as significant damage to the molecular structure of the fullerenes, which is comparable with the XRD analysis results.

### Investigation of phase fraction in Al/C composites

**Figure 5:**
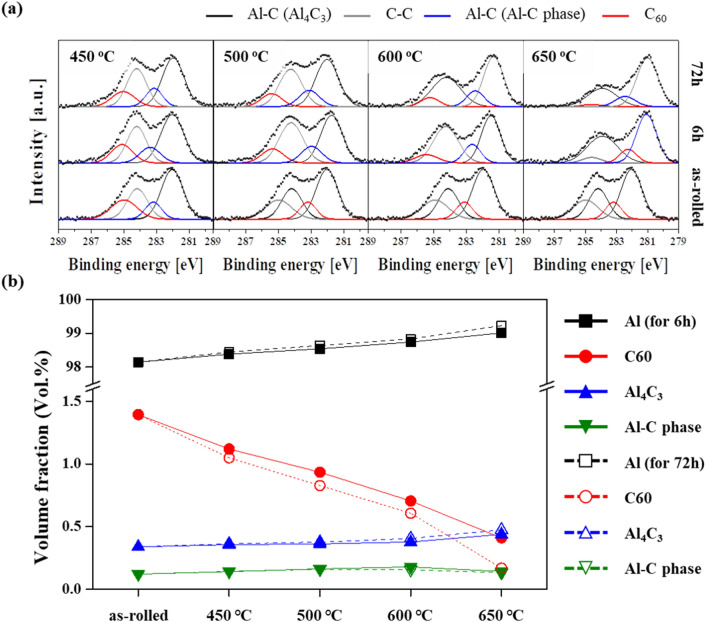
(**a**) XPS spectra (C1s) of Al/C60 nanocomposites after heat treatment at various temperatures for 6 and 72 h, and (**b**) the resulting variation of phase fraction.

In order to measure the fraction of each phase (i.e., Al matrix, C_60_, Al_4_C_3_, and supersaturated Al-C phases), X-ray photoelectron spectroscopy (XPS) analysis is conducted on the composites under various heat-treatment conditions. Figure 5a shows XPS spectra of the C1s region of Al/C composites heat-treated for 0, 6, and 72 h under 450, 500, 600, and 650 °C. The spectra include peaks corresponding to C–C in adventitious carbon (~ 284.2 eV), C–C in fullerene (~ 285.4 eV), Al-C in Al_4_C_3_ (~ 282.0 eV), and Al-C in the Al-C phase (~ 283.2 eV)^21^. After separation of the peaks, we calculate the area fraction of the C_60_, Al_4_C_3_, and Al-C phases, as summarized in Table 1. Because the area fraction is equivalent to the sum of the atomic ratio of the carbon atoms contained in the phases^33^, the volume fraction of the Al, C_60_, Al_4_C_3_, and Al-C phases can be estimated from the atomic fraction using the physical parameters of the phases (i.e., density and molar weight) and with assumption that the carbon sources containing the carbide and Al-C phase are induced from only fullerenes. Consequently, Fig. 5b presents the evolution of the volume fraction of the phases. For the as-rolled Al/C composites, the measured volume fraction of Al and C_60_ differs from the designed fraction. This might be related to the formation of the Al_4_C_3_ and Al-C phases during the hot-rolling process carried out at 480 °C and the large difference in molar weight. Phase formation is affected by heat treatment; the temperature range used in this process determines which phase will be formed. The volume fractions of the Al_4_C_3_ and Al-C phases tend to increase with a decreasing C_60_ fraction after heat treatment at the various temperatures for 6 h, and the fraction of the Al-C phases in the Al/C composite heat-treated at 450 and 500 °C is similar to the fraction in the 72-h-treated composite. This may indicate that the supersaturated Al-C phase is thermally stable below 500 °C. At 600 °C, the fraction of Al-C phases is reduced by increasing the heat treatment duration from 6 to 72 h, while the fraction of the Al_4_C_3_ phase increases more. The Al-C phases are transformed into carbide after heat treatment at 600 °C for a longer duration. Finally, Al_4_C_3_ is primarily formed at 650 °C.

**Table 1:** Summary of area fraction of phases observed in Al/C composites.

Phase	Area fraction [%]								
	as rolled	450 °C		500 °C		600 °C		650 °C	
		6 h	72 h	6 h	72 h	6 h	72 h	6 h	72 h
C_60_	26.92	21.58	20.20	17.97	15.89	13.52	11.64	7.85	3.21
Al_4_C_3_	58.04	60.92	62.11	61.78	64.28	64.38	69.07	74.63	80.54
Al-C phase	15.04	17.50	17.69	20.25	19.82	22.10	19.30	17.53	16.25

### The evolution in elastic and plastic behavior of Al/C composites

**Figure 6:**
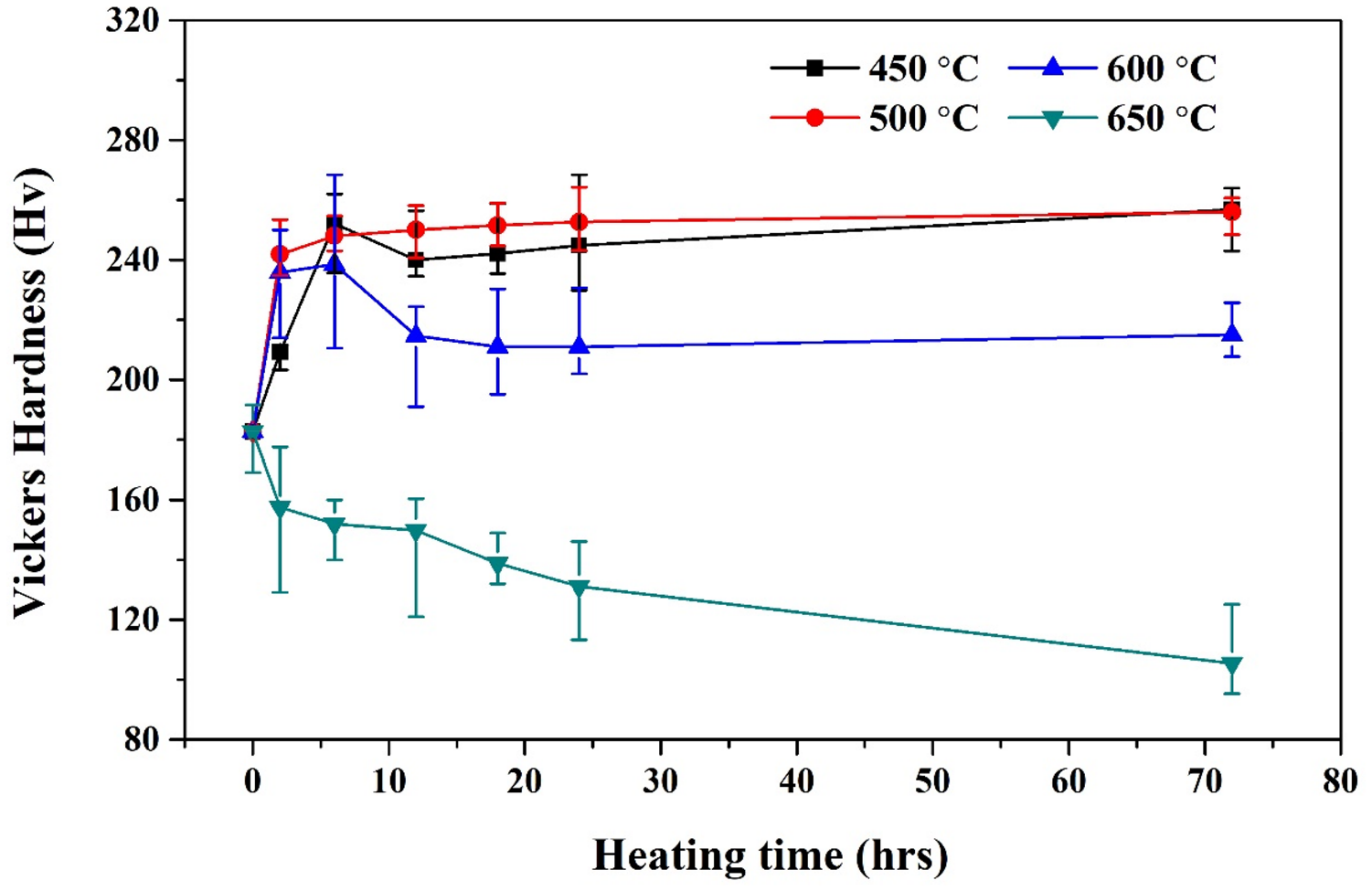
Vickers hardness variation of Al composites depending on heat treatment temperatures and times.

Figure 6 reveals the Vickers hardness of the composites heat-treated at various temperatures as a function of the heat-treatment duration. While the initial Vickers hardness of the Al-fullerene composite is 182.72 Hv, the hardness of the composites heat-treated at 450 and 500 °C for 6 h significantly increased to 252.12 and 248.10 Hv, respectively. Decomposition of fullerenes and the formation of Al-C phases appear to increase hardness. Load transfer from the matrix to the fullerenes might be insignificant because of the spherical shape of the fullerenes with an extremely low aspect ratio. Hence, the strengthening of the fullerenes is not expected to be critical, although they have high strength stemming from the strong covalent bonds among their carbon atoms. These nano-scale particles mainly increase the strength of the Al matrix by impeding dislocation motion (i.e., Orowan strengthening). In this regard, the semi-coherent interface between the Al-C phases and the Al matrix can block dislocation motion and consequently increase the hardness of the composites compared to the incoherent interface between the fullerenes and the Al matrix. Moreover, their hardness gradually increases to 256.92 and 255.98 Hv, respectively, upon heating for 72 h. Although Al_4_C_3_ is formed in Al/C composites, the nano-scale Al carbides are also considered to lead to enhancement of the hardness of the composites. However, the hardness of the Al/C composites heat-treated at 600 °C changes depending on the heat-treatment duration; whereas the hardness increases to 238.56 Hv after heat treatment for 6 h, it decreases to 215.02 Hv after 72 h. For 650 °C heat-treated Al/C composites, the hardness decreases with increasing treatment time. The decrease in hardness of the composites might be related to both coarsening of the carbides and/or grain growth of the Al matrix during the heat treatment.

**Figure 7:**
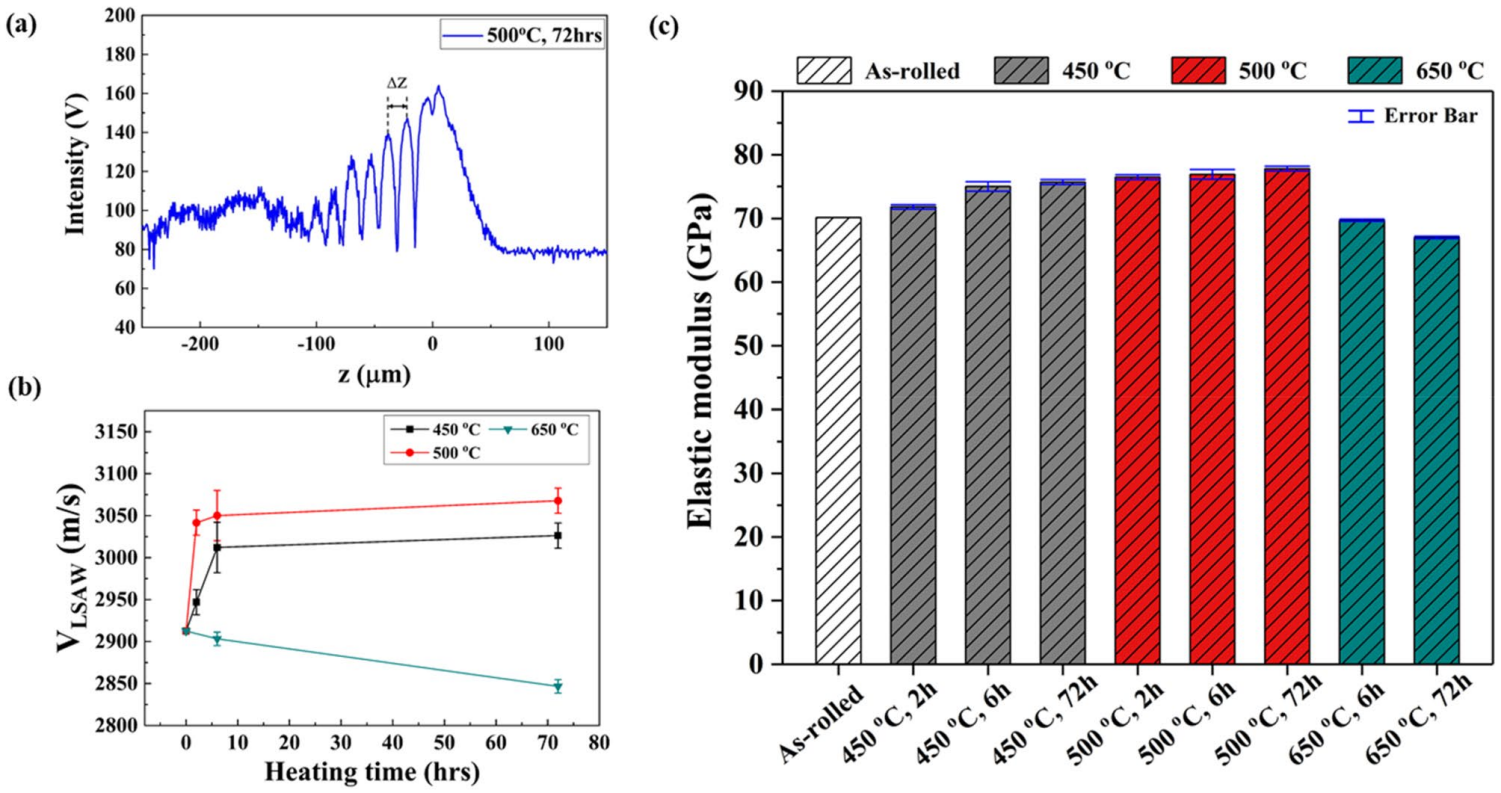
(**a**) V(z) curve for Al-C composite specimen heat-treated at 500℃ for 72 h, (**b**) Leaky surface acoustic wave velocity, *v*_*LSAW*_, for Al-C composite specimens as functions of heat treatment time and temperature, and (**c**) variation of elastic modulus for Al-C composite specimens as functions of heat treatment time (h) and temperature.

In order to characterize the acoustic and mechanical properties of the heat-treated Al/C composites, we obtained their V(z) curves for Al/C composites heat treated at 450, 500, and 650 °C for different hours (2, 6, 72 h). Figure 7a depicts the experimental V(z) curve for a Al/C composite specimen annealed at 500 °C for 72 h. A strong central maximum is clearly observed at the focal plane, z = 0. This arises from the primary reflection, which does not depend on the material properties of the specimen being measured. When z becomes negative, as shown in the left side of Fig. 7a, a series of oscillations with periodic maxima and minima take place, from which a characteristic period Δz can be estimated. In the specimen annealed at 500 °C for 72 h, Δz is 14.62 μm. From this, the LSAW velocity is calculated as 3067.8 m/s, according to Eq. (1). Similar oscillating behaviors are observed for other Al/C composite specimens; however, each exhibits different characteristic Δz values depending on the heat treatment conditions.

Figure 7b presents the LSAW velocities estimated from the V(z) curves of the Al/C composites heat-treated at various temperatures for different duration times. In general, acoustic wave velocity is known to be a function of the elastic properties, mass density, microstructure, and other parameters of the studied material^34^. Here, the measured LSAW velocities of the Al/C composite specimens under various heating conditions provide experimental support for their dependence on the microstructural characteristics, which are thoroughly observed in the XRD patterns, Raman spectra, and XPS results.

First, the LSAW velocities of the Al/C composites range from 2846. 6 m/s to 3067.8 m/s, exhibiting apparent dependence on the heat-treatment conditions. The as-rolled Al-C_60_ composite is shown to have an LSAW value of 2912.5 m/s. These measured surface acoustic wave velocities of the Al/C composites appear to fall within a reasonable range of expectation, particularly considering that the longitudinal and shear acoustic velocities of aluminum are approximately 6375 m/s and 3130 m/s, respectively^35^, and that the surface acoustic wave velocity of a material is generally expected to be approximately 90% of the shear acoustic velocity value for that material.

Second, Fig. 7b shows that differences in heat treatment temperatures result in different acoustic properties of the Al/C composite specimens. For instance, heat treatment at 450 and 500 °C increases the LSAW velocity compared to the as-rolled specimen, whereas heat treatment at 650 °C decreases the LSAW velocity of the Al/C composites. Microstructural changes upon heating such as fullerene decomposition and the formation of Al-C supersaturated phases and/or Al_4_C_3_ carbides result in either faster or slower propagation of the acoustic waves on the surface of the Al/C composites depending on the annealing temperatures. As indicated by the XPS spectra shown in Fig. 5a, relatively more Al-C supersaturated phases are formed in the Al/C composites annealed at 450 and 500 °C than at other temperatures, contributing to faster acoustic wave propagation in these specimens. In contrast, the formation of a significant fraction of Al_4_C_3_ appears to cause surface acoustic waves to propagate more slowly in the Al/C composite specimens heat-treated at 650 °C than in the as-rolled specimens.

Additionally, the heating duration time affects the resulting LSAW velocities of the Al/C composites. It is clearly observed in Fig. 7b that the degree of increase in the LSAW velocity varies depending on the duration time. The Al/C composite specimens annealed at 450 °C, for instance, exhibit a substantial increase in the first 6 h of the heat treatment. After that point, the LSAW velocity increases at a much slower rate as a function of the heating time. For specimens annealed at 500 °C, the change in the LSAW velocity is relatively less sensitive to the heating time than in those annealed at 450 °C. This is likely because higher temperature provides more kinetic energy relatively faster and thus results in faster microstructural and phase changes in the specimens, which affects the acoustic propagation properties of those specimens (Fig. 7b).

We estimated the elastic modulus in each Al/C composite specimen using the following relationship between the surface acoustic wave velocity *v*_*LSAW*_ and the bulk longitudinal and transverse sound velocity, *v*_*l*_, *v*_*t*_^34,36^:

1 $$ v_{{LSAW}}  = \frac{{v_{0} }}{{\left\{ {1 - \left( {1 - \frac{{v_{0} }}{{2f\Delta z}}} \right)^{2} } \right\}^{{1/2}} }} $$

2 $$ \left( {\frac{{v_{{LSAW}} }}{{v_{t} }}} \right)^{6}  - 8\left( {\frac{{v_{{LSAW}} }}{{v_{t} }}} \right)^{4}  + 8\left( {\frac{{v_{{LSAW}} }}{{v_{t} }}} \right)^{2} \left\{ {3 - 2\frac{{v_{t} ^{2} }}{{v_{l} ^{2} }}} \right\} - 16\left\{ {1 - \frac{{v_{t} ^{2} }}{{v_{l} ^{2} }}} \right\} = 0 $$

where Δz is the oscillation interval in the V(z) curve, *v*_*0*_ is the velocity of the acoustic waves in the coupling fluid, and *f* is the frequency (i.e., 400 MHz in this case). Moreover, the bulk longitudinal and transverse sound velocity, *v*_*l*_ and *v*_*t*_ , respectively, are related to the material’s elastic properties including its elastic modulus (*E)*, Poisson’s ratio (*ν)*, and mass density (*ρ)* according to the following Eqs. (3a) and (3b):

3a $$ v_{l}  = \sqrt {\left( {\frac{{E\left( {1 - v} \right)}}{{\rho \left( {1 + v} \right)\left( {1 - 2v} \right)}}} \right)} $$

3b $$ v_{t}  = \sqrt {\left( {\frac{E}{{\rho 2\left( {1 + v} \right)}}} \right)} $$

Rearranging Eq. (3) gives an expression of the elastic modulus E as well as the ratio of the bulk transverse to the bulk longitudinal sound velocity:

4a $$ E = \frac{{\rho v_{t} ^{2} \left( {3v_{l} ^{2}  - 4v_{t} ^{2} } \right)}}{{(v_{l} ^{2}  - v_{t} ^{2} )}} $$

4b $$ \frac{{v_{t} }}{{v_{l} }} = \sqrt {\left\{ {\frac{{\left( {1 - 2v} \right)}}{{2\left( {1 - v} \right)}}} \right\}} $$

Assuming that Poisson’s ratio and the density of the Al/C composites are 0.33 and 2700 g/m^3^, respectively, we can calculate the elastic modulus E using Eqs. (2)–(4) and measured *v*_*LSAW*_ data from SAM. The resulting elastic moduli of the Al/C composites under various heating-treatment conditions are presented in Fig. 7c. The elastic modulus of the as-rolled Al/C composite specimen is determined to be 70.1 GPa, and it increases or decreases depending on the heating temperature and duration, ranging from 67.0 to 77.8 GPa.

## Discussion

For pure Al, the elastic modulus and Vickers hardness are measured to be 70.1 GPa and 26 Hv, respectively. Although the modulus of pure Al is similar to that of the as-rolled specimen, the hardness significantly differs owing to the strengthening by grain refinement. In this study, we focus on the contribution of C_60_, Al_4_C_3_, and supersaturated Al-C phases to the improvement of elastic and plastic behaviors in Al/C composites, except for the grain size effect. Hence, the evolution of properties was investigated after heat treatment of the as-rolled specimens.

The elastic property of a material is the result of its elastic response to mechanical forces (e.g., acoustic waves)—dimensional changes that correspond to departures of interatomic spacings from equilibrium spacing on the atomic level. Thus, the relative fractions of phases that have different atomic bonding in each specimen are expected to contribute to the elastic property differently. It is possible to categorize our Al-C specimens into the following four groups, each containing different microstructural phases and corresponding atomic bonding, mostly depending on the heat-treatment temperature. First, the as-rolled Al-C specimen is a composite simply consisting of Al-Al metal bonding in the Al matrix and C–C covalent bonding in the fullerenes. The elastic modulus of and the as-rolled Al/C composite specimen is 70.1 GPa. The second group includes the specimen heat-treated at 450 °C, in which the formation of the supersaturated Al-C phases started while the Al-C_60_ composite phases were still dominant. The presence of supersaturated Al-C phases appears to lead to a slight increase in the elastic modulus to 71.8 ~ 75.7 GPa, when compared to the as-rolled specimen. Third, the specimens heat-treated at 500 °C demonstrate a dominant fraction of supersaturated Al-C phases. In these specimens, a reduction in the composite phases is also confirmed by the Raman spectra in Fig. 4b, where broader and lower peaks corresponding to C–C bonding in the fullerenes suggest partial destruction of the fullerenes’ molecular structure and the formation of further Al-C supersaturated phases. It should be noted here that the maximum elastic modulus of 77.8 GPa is found in the Al/C composite specimen heat-treated at 500 °C for 72 h, which also demonstrates the maximum Vickers hardness (Fig. 6). Finally, the last group includes the specimen heat-treated at 650 °C, which undergoes a reduction in the elastic modulus, where the formation of carbide Al_4_C_3_ phases becomes dominant and most fullerenes seem to disappear, as observed through the XRD, Raman, and XPS analyses.

To summarize, the elastic modulus results in Fig. 7c lead to the following conclusions. (1) It is possible to control the elastic property by varying the temperature and duration of the heat treatment. (2) Depending on the heat-treatment conditions, either a decrease or an increase of the elastic modulus can occur. For Al/C composites, 500 °C is the optimal heat-treatment temperature for obtaining the maximum elastic modulus and hardness. (3) The degree of change in the elastic modulus is not as substantial as the variation of the Vickers hardness upon heat treatment. The maximum hardness is about 255.98 Hv when the specimen is annealed at 500 °C for 72 h, which is 40.1% larger than that of the as-rolled specimen (182.72 Hv). In contrast, the maximum elastic modulus achieved is 77.8 GPa, which is increased by 10.9% compared to that of the as-rolled specimen (70.1 GPa). This implies that the microstructural evolution of the various phases, including the Al matrix, the nano-carbon reinforcement, the Al carbide, and the Al-C phases, upon heat treatment differently affects the elastic and plastic properties of Al/C composites.

**Figure 8:**
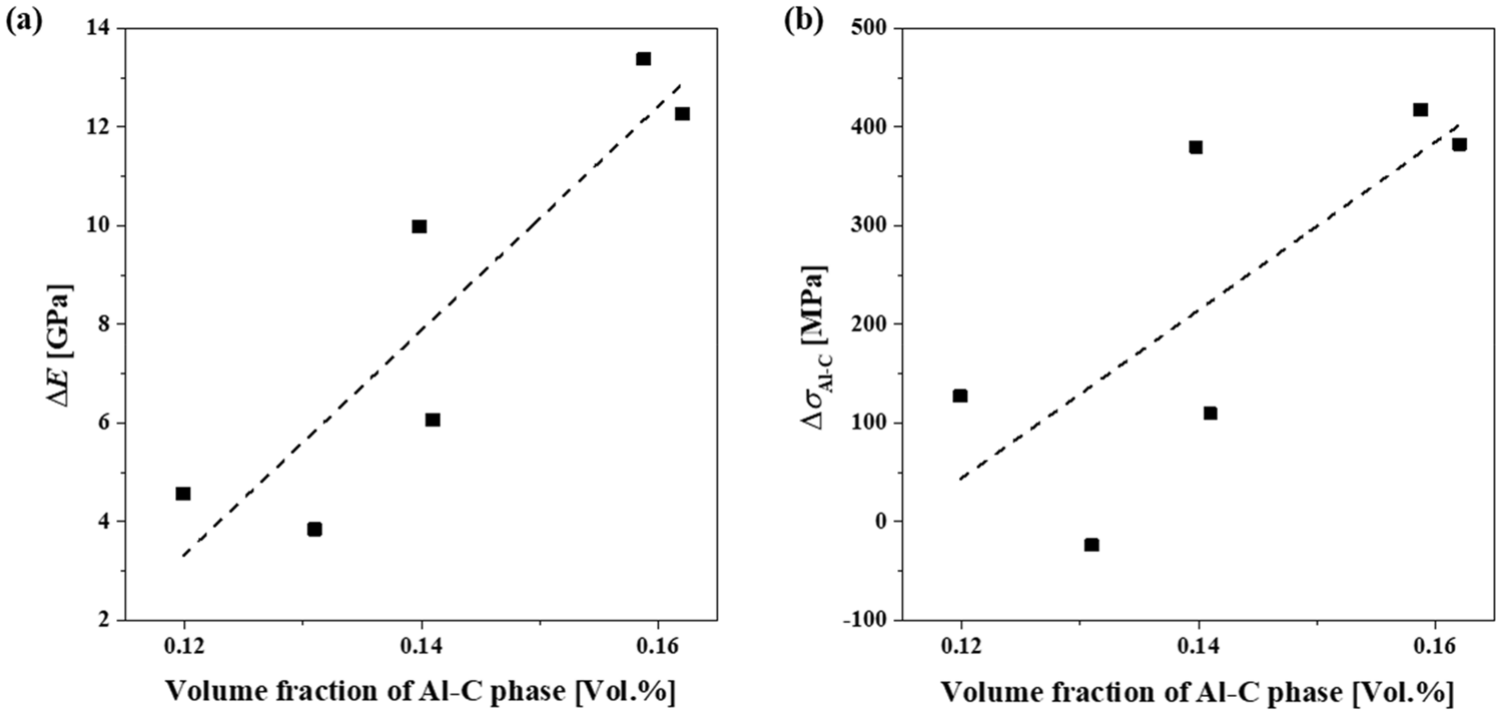
Variation of the difference in (**a**) elastic modulus and (**b**) hardness as a function of volume fraction of Al-C phase.

In order to compare the contribution of C_60_, Al_4_C_3_, and supersaturated Al-C phases to the enhancement of the elastic modulus, the theoretical elastic modulus of the Al/C composites is calculated using the rule of mixture, as expressed below:

5 $$ E_{{compo}}  = E_{{Al}}  \cdot V_{{Al}}  + ~K_{{C60}}  \cdot E_{{C60}}  \cdot V_{{C60}}  + ~K_{{Al4C3}}  \cdot E_{{Al4C3}}  \cdot V_{{Al4C3}}  + ~K_{{Al - C}}  \cdot E_{{Al - C}}  \cdot V_{{Al - C}} $$

where *E*_*compo*_ is the elastic modulus of the Al/C composite; *E*_*phase*_, *V*_*phase*_, and *K*_*phase*_ are the elastic modulus, volume fraction, and reinforcing coefficient of the phases observed in the composite, respectively. According to the reported literature, the elastic modulus of Al, C_60_, and Al_4_C_3_ is assumed to be 70, 860, and 216 GPa, respectively^9,37,38^. Moreover, the reinforcing coefficient of C_60_ may be presumed to be 0.5, wherein the molar structure is damaged during the ball-milling process^39^. Because the Al_4_C_3_ morphology is similar to that of a short fiber, its coefficient is determined to be 0.2 in this study^40^. The volume fraction of each phase is shown in Fig. 5b. The contribution of the Al-C phases to the increase of the elastic modulus of the Al/C composites ($E^{\prime}_{{Al - C}} ;~E^{\prime}_{{Al - C}}  = K_{{Al - C}}  \cdot E_{{Al - C}}$) thus can be estimated from the slope in the graph of the difference between the measured and calculated modulus (i.e., $\Delta E = ~E_{{compo}}^{{measured}}  - E_{{Al}}  \cdot V_{{Al}}  - ~K_{{C60}}  \cdot E_{{C60}}  \cdot V_{{C60}}  - ~K_{{Al4C3}}  \cdot E_{{Al4C3}}  \cdot V_{{Al4C3}}$) *Vs* the volume fraction of the Al-C phases (Fig. 8a). Consequently, the Al-C phases have a significantly higher contribution to the enhancement of the elastic modulus of Al/C composites of approximately 227 GPa/vol%. The reinforcing effect of the Al-C phases is found to be significantly greater than that of fullerenes (i.e., 2.58 GPa/vol%) or aluminum carbides (i.e., 0.43 GPa/vol%). Similarly, we calculate the contribution of phases on the hardness of the Al/C composites ($\sigma _{{compo}}  = \sigma _{{Al}}  \cdot V_{{Al}}  + ~K_{{C60}}  \cdot \sigma _{{C60}}  \cdot V_{{C60}}  + ~K_{{Al4C3}}  \cdot \sigma _{{Al4C3}}  \cdot V_{{Al4C3}}  + ~K_{{Al - C}}  \cdot \sigma _{{Al - C}}  \cdot V_{{Al - C}}$). When the strength of the Al/C composites is estimated based on the relationship *σ* =3.3 *Hv* (where *Hv* is the Vickers hardness)^41^ and the strength of Al, fullerene, and Al_4_C_3_ was assumed to be 0.35, 30, and 10 GPa, respectively^9,38,42^, the reinforcing contribution of fullerenes, carbides, and the Al-C phases becomes 9, 2, and 851 GPa/vol% (Fig. 8b), respectively. Given that covalent bonds in fullerenes or ionic bonds in carbides are much stronger than metallic bonds that may be present in the Al-C phases, it is very interesting that the Al-C phases have the highest reinforcing effect on the elastic modulus and hardness. This is possibly because the carbon atoms, which are well dispersed on the atomic scale, may greatly alter the bonding characteristics of the metallic bonds between the aluminum atoms by (i) significantly changing the atomic distance of the aluminum atoms and/or (ii) changing the metallic bonding features to ionic bonding features^43^. Furthermore, the distorted lattice structure of the Al-C phases hinders the dislocation movement, thereby improving the strength of the Al/C composites. Hence, we attribute the enhancement of the mechanical properties of the heat-treated Al/C composites to the formation of the Al-C phases.

## Conclusion

We manipulated the fractions of C_60_-fullerenes, nano-scale carbides, and Al-C supersaturated phases in aluminum matrix composites by annealing Al/C_60_ composites. Furthermore, the contribution of each phase to the elastic and plastic behaviors of the composites was examined through the SAM technique and hardness measurements. After heat treatment, supersaturated Al-C phases and Al carbide were formed in the Al/C composites by decomposition of individually dispersed C_60_. As a result, the hardness and the elastic modulus of the Al/C composites were enhanced during the heat treatment. The hardness of the Al/C composite slightly increased from 182.7 Hv to approximately 250 Hv after heat treatment at 450 and 500 °C for 72 h. However, the hardness of the 650 °C heat-treated composites was reduced to 105.4 Hv. Similar results were produced for the elastic property; the elastic modulus of the 500 °C heat-treated Al/C composites was significantly enhanced from 70.1 to 77.8 GPa, but the value decreased to 67.0 GPa after heat treatment at 650 °C. This is because the Al-C phases significantly enhance the elastic modulus and the hardness of Al/C composites to 227 GPa/vol% and 851 GPa/vol%, respectively. We attribute this phenomenon to the well-dispersed C on the atomic scale changing the elastic bonding characteristics of the metallic bonds between the Al atoms and blocking dislocation movement in the Al/C composites.

## Methods

Al/C composites were fabricated through hot rolling followed by heat treatment using three-step ball-milled powders from pure Al powder and fullerenes. Figure 2 shows a schematic representation of the experimental procedures for synthesizing the Al/C composites. An Al-based composite powder containing 5 vol% fullerene was prepared through three-step ball-milling. First, the pristine fullerenes (99.5% purity, SES Research Co., USA) were shattered using a planetary mill (Pulverisette 5, Frisch, Germany) in order to break the van der Waals bonds in the fullerenes. Fullerenes (2.5 g) and stainless-steel balls (1200 g) with 5 mm diameter were placed in a stainless-steel chamber. To prevent excessive cold-welding of the powders, 1 wt% stearic acid (CH_3_(CH_2_)_16_COOH, Sigma Aldrich Korea Co, Ltd, Korea) was added as a process control agent in the chamber. We then performed eight cycles of milling at 200 rpm; each cycle lasted 15 min and it was followed by a pause of 75 min. Afterward, pure Al powder (99.5% purity, Changsung Co., Ltd., Korea) was added in the shattered fullerene; the fullerene was pre-mixed with 77.5 g of Al powder via planetary milling under the same conditions as in the previous step. The composition of Al powder as starting powder was summarized in Table 2. Finally, fullerene-dispersed Al composite powder was prepared via high-energy ball-milling using an attrition mill (KMC-1BV, KMC Co. Ltd., Korea). The mixed powder and stainless-steel balls with a ball-to-powder ratio of 15:1 was placed into the stainless-steel chamber. Stearic acid (1 wt%) was added in the chamber. To prevent an increase of the internal temperature during ball-milling, cooling water was circulated around the walls of the chamber. Attrition milling was then conducted at 500 rpm for 24 h in an argon atmosphere.

**Table 2:** Chemical composition of Al powder as starting material.

Fe	Ti	Cr	Ni	Mn	Cu	Si	O	Al
0.025	0.056	0.044	0.082	0.008	0.006	0.096	0.013	Bal

The Al-fullerene composite powder was fully consolidated by hot rolling. The ball-milled powder was placed in a one-side-sealed copper tube (60 mm in diameter, 150 mm in height), and then the tube was sealed. Hot rolling was carried out at 480 °C with 12% reduction per pass until the sample thickness reached 1.23 mm. After peeling off the copper container, the sample was heat-treated at 450, 500, 600, and 650 °C for 6 and 72 h in order to investigate mechanical properties of the Al/C composites, which depend on their microstructural evolution, wherein the Al-C phases and the nano-size Al_4_C_3_ phase were formed during the heat treatment.

The morphology of the powder at each step of the ball-milling process was observed using a SEM (JEM 7610F, JEOL, Japan), and the microstructures of the Al/C composites were observed using TEM (Technai G2 F20, FEI, USA). The phases of the Al/C composites were identified through XRD (CN2310, Rigaku, Japan) with Cu Ka radiation. Raman spectroscopy (LabRam Aramis, Horiba Jobin Yvon Co. Ltd., France) was used to investigate the changing molecular structure of the fullerenes and the formation of carbides during the heat treatment. XPS (K-alpha, Thermo, USA) was carried out in the Al2p region in order to quantify the fraction of phases observed in the Al/C composites. The hardness variation depending on the heat treatment condition was measured using a micro-Vickers hardness tester (HM 211, Mitutoyo, Japan) under an applied load of 300 g.

Acoustic measurements were conducted using a SAM (UH-3, Olympus, Japan) equipped with a 400-MHz acoustic lens (AL4M631). Distilled water was used as a coupling fluid at room temperature. V(z) measurements were performed with z values ranging from − 250 μm to 150 μm in the sub-surface, where z was set to zero at the focal point. In the present work, the velocity of the acoustic waves in distilled water was 1505.9 m/s. The variation of Young’s modulus as a function of the heat-treatment conditions could then be estimated from the LSAW velocity. The experiments were repeated at least five times to obtain an average of the results.
